# Significance of nicotine and nicotinic acetylcholine receptors in Parkinson’s disease

**DOI:** 10.3389/fnagi.2025.1535310

**Published:** 2025-03-21

**Authors:** Xia Lin, Qian Li, Min Pu, Hao Dong, Qinghua Zhang

**Affiliations:** ^1^Department of Neurology, First People's Hospital of Tianshui, Tianshui, Gansu, China; ^2^First Clinical Medical School, Gansu University of Chinese Medicine, Lanzhou, Gansu, China

**Keywords:** Parkinson’s disease (PD), neurodegeneration, nicotine, nicotinic acetylcholine receptors, *α*-synuclein

## Abstract

Parkinson’s disease (PD) is a multifaceted neurodegenerative disorder characterized by the degeneration of dopaminergic neurons in the substantia nigra and the aggregation of *α*-synuclein. According to epidemiological data, PD is the second most prevalent neurodegenerative disorder after Alzheimer’s disease (AD) and has emerged as a significant global health concern. This review examines the intricate pathological mechanisms and high-risk factors associated with PD, and discusses the challenges in its clinical diagnosis and treatment. We elucidate the relationship between smoking and the reduced risk of PD, highlighting the potential neuroprotective effects of nicotine present in tobacco. The interaction between nicotine and nicotinic acetylcholine receptors (nAChRs) is analyzed in detail, emphasizing their neuroprotective capabilities and underlying molecular mechanisms. Furthermore, we analyze the structural and functional diversity of nAChRs and their roles in the pathological progression of PD. Our review aims to elucidate the complex interplay of genetic, environmental, and biochemical factors in PD and to propose future research directions that may facilitate therapeutic development.

## 1 Introduction

Parkinson’s disease (PD) is a neurodegenerative disorder that primarily affects the elderly population, characterized by the progressive degeneration of dopaminergic neurons in the substantia nigra and the formation of pathological *α*-synuclein aggregates, known as Lewy bodies. The clinical manifestations of PD include a range of motor symptoms, such as tremors and rigidity, as well as non-motor symptoms, including olfactory dysfunction and depression. The onset of PD is the result of a complex interplay of factors, including genetic susceptibility and environmental exposures (Ben-Shlomo et al., 2024). Despite some progress in understanding the pathogenesis of Parkinson’s disease (PD), clinical diagnosis and treatment remain suboptimal, facing significant challenges such as disease heterogeneity.

Epidemiological studies have revealed an intriguing observation: the incidence of PD is lower among smokers. This finding has prompted research into the active components in tobacco that might effectively intervene in the progression of the disease (Rose et al., 2024). Although smoking has negative effects on neural structure, neurotransmission, and cognitive function, multiple studies have shown that smoking is associated with a lower risk of Parkinson’s disease (PD). This protective effect even extends to the pathological progression of PD and its corresponding incidence (Hernan et al., 2002). Nicotine is a naturally occurring alkaloid primarily found in tobacco plants. As the main component of tobacco, it is associated with stimulation of the central nervous system and is linked to cardiovascular diseases and various cancers. However, nicotine is also an agonist of nicotinic acetylcholine receptors (nAChRs), playing a significant regulatory role in neurotransmitter release, neuronal excitability, and cognitive function (Ascherio and Schwarzschild, 2016; Dineley et al., 2015). Therefore, nicotinic acetylcholine receptors (nAChRs), which are targets of nicotine, have garnered attention for their potential neuroprotective effects. These receptors play a critical role in neuronal communication and regulation, and they may play an important role in modulating the pathology of Parkinson’s disease (PD) (Quik et al., 2008).

This review aims to elucidate the multifactorial nature of Parkinson’s disease (PD) by exploring the potential protective mechanisms of smoking and the therapeutic prospects of targeting nicotinic acetylcholine receptors (nAChRs). By synthesizing current research, we hope to provide insights into the foundational mechanisms of PD, thereby linking tobacco-based research to potential disease intervention pathways and offering inspiration for the clinical development of PD treatments.

## 2 The pathophysiology of Parkinson’s disease

Parkinson’s disease, first described by James Parkinson in 1817, is a complex, age-related, progressive, and multisystem neurodegenerative condition associated with dopaminergic neuronal loss and formation of *α*-synuclein (α-Syn) protein aggregation in neurons of the substantia nigra (SN) (Ben-Shlomo et al., 2024). Specifically, loss of dopaminergic neurons that project from the SN pars compacta to the caudate-putamen in the striatum leads to the impairment of dopamine neurotransmission, eliciting various clinical syndromes, including pathological motor features, such as a slowly progressive asymmetric resting tremor (bradykinesia), cogwheel rigidity, and gait disturbance, as well as non-motor features, such as anosmia, constipation, depression, sleep behavior disorder, autonomic dysfunction, pain, and psychiatric symptoms (Morris et al., 2024). Under physiological conditions, *α*-Syn, a 140-amino-acid protein, consisting of amphipathic alpha-helical repeats and encoded by the SNCA gene, is an abundant neuronal protein enriched at presynaptic terminals of neurons, mediating neurotransmission through binding to synaptic vesicles via an N-terminal membrane-binding region (Sharma and Burré, 2023). Pathologically, *α*-Syn is subjected to a conformational change from *α*-helical state to *β*-sheet-rich oligomers and fibrils capable of aggregation due to mutations in its encoding gene SNCA, altering SNCA gene transcription and mRNA stability or processing (Nalls et al., 2019; Blauwendraat et al., 2019). Moreover, several post-translational modifications of *α*-Syn also contribute to its aggregation, such as phosphorylation at residues Y125, Y133, Y136, S87, and S129 (Sharma and Burré, 2023; Fujiwara et al., 2002). Aggregated α-Syn proteins then act as a major component of Lewy bodies and Lewy neurites which are typical hallmarks of PD (Wilson 3rd et al., 2023). Significantly, Lewy bodies contain hundreds of other proteins and dysmorphic organelles, including lysosomes and mitochondria, packaged by abundant lipid membranes (Mahul-Mellier et al., 2020). Lewy bodies have detrimental effects on neurons because they are space-occupying lesions that have the potential to change cellular function, and studies suggest that small aggregates and oligomers of Lewy bodies are more toxic than larger ones (Alam et al., 2019). In addition, it has been reported that approximately 90 distinct genes across 74 genomic loci are associated with PD susceptibility (Blauwendraat et al., 2020). We have summarized risk genes strongly correlated with the risk of PD in Table 1. Notably, it has been identified that age at onset and extreme or typical phenotypes of PD are associated with polygenic rather than monogenic, and single-gene variants, including high-penetrance, mendelian alleles, are only discovered in 5–10% of all PD cases (Ye et al., 2023). Meanwhile, it has been reported that other pathological underlying causes, such as typical Alzheimer’s disease (AD) and Huntington’s disease (HD) pathology, including type 2 microtubule-associated protein (Tau) aggregation, and formation of polyglutamine might contribute to the onset of PD (Geddes et al., 1993; Kalia et al., 2015; Doherty et al., 2013).

**Table 1:** The high-risk genes in PD.

Previously identified gene	The nearest gene
KRTCAP2	FCGR2A
STK39	VAMP4
TMEM175	KCNS3
SNCA	LINC00693
LRRK2	SPTSSB
CRHR1	KCNIP3
MCCC1	MED12L
NUCKS1	KPNA1
BST1	LCORL
HLA–DRB5	CLCN3
GPNMB	RIMS1
INPP5F	PAM
LRRK2	C5orf24
GBA	RIMS1
RAB29	RPS12
GAK	FYN
ELOVL7	TRIM40
RIT2	GS1–124 K5·11
SPPL2B	UBAP2
SH3GL2	GBF1
IGSF9B	RNF141
FGF20	FAM49B
ITPKB	SCAF11
PMVK	FBRSL1
MAP4K4	MBNL2
SATB1	RPS6KL1
IP6K2	CAB39L
FAM47E	CD19
SCARB2	MIPOL1
CAMK2D	CHRNB1
LOC100131289	UBTF
CTSB	NOD2
FGF20	GRN
FBIN3	MED13
SH3GL2	MEX3C
ITGA8	ASXL3
BAG3	DYRK1A
DLG2	DNAH17
IGSF9B	CRLS1
GCH1	
CHD9	
GALC	
VPS13C	
CASC16	
SYT17	
CRHR1	
RETREG3	
WNT3	

The gene screening data were derived from the exploration of public databases. These data were obtained through a meta-analysis of a large-sample database, specifically the PD meta-GWAS. This analysis assessed the risk correlation coefficients of genes associated with Parkinson’s disease by evaluating indicators such as gene expression levels, mutation rates, and methylation patterns (Nalls et al., 2019). KRTCAP2, Keratinocyte Associated Protein 2; STK39, Serine/Threonine Kinase 39; TMEM175, Transmembrane Protein 175; SNCA, Synuclein Alpha; LRRK2, Leucine Rich Repeat Kinase 2; CRHR1, Corticotropin Releasing Hormone Receptor 1; MCCC1, Methylcrotonoyl CoA Carboxylase 1; NUCKS1, Nuclear Casein Kinase and Cyclin Dependent Kinase Substrate 1; BST1, Bone Marrow Stromal Cell Antigen 1; HLA DRB5, Major Histocompatibility Complex Class II DR Beta 5; GPNMB, Glycoprotein Nmb; INPP5F, Inositol Polyphosphate 5 Phosphatase F; GBA, Glucosylceramidase Beta; RAB29, RAB29 Member RAS Oncogene Family; GAK, Cyclin G Associated Kinase; ELOVL7, ELOVL Fatty Acid Elongase 7; RIT2, Ras Like Without CAAX 2; SPPL2B, Signal Peptide Peptidase Like 2B; SH3GL2, SH3 Domain Containing GRB2 Like 2 Endophilin A1; IGSF9B, Immunoglobulin Superfamily Member 9B; FGF20, Fibroblast Growth Factor 20; ITPKB, Inositol Trisphosphate 3 Kinase B; PMVK, Phosphomevalonate Kinase; MAP4K4, Mitogen Activated Protein Kinase 4; SATB1, SATB Homeobox 1; IP6K2, Inositol Hexakisphosphate Kinase 2; FAM47E, Family With Sequence Similarity 47 Member E; SCARB2, Scavenger Receptor Class B Member 2; CAMK2D, Calcium Calmodulin Dependent Protein Kinase II Delta; LOC100131289, Uncharacterized LOC100131289; CTSB, Cathepsin B; FBIN3, F Box And WD Repeat Domain Containing 7; ITGA8, Integrin Subunit Alpha 8; BAG3, BCL2 Associated Athanogene 3; DLG2, Discs Large MAGUK Scaffold Protein 2; GCH1, GTP Cyclohydrolase 1; CHD9, Chromodomain Helicase DNA Binding Protein 9; GALC, Galactosylceramidase; VPS13C, Vacuolar Protein Sorting 13 Homolog C; CASC16, Cancer Susceptibility Candidate 16; SYT17, Synaptotagmin 17; RETREG3, Reticulophagy Regulator Family Member 3; WNT3, Wnt Family Member 3; FCGR2A, Fc Fragment Of IgG Receptor IIa; VAMP4, Vesicle Associated Membrane Protein 4; KCNS3, Potassium Voltage Gated Channel Modifier Subfamily S Member 3; LINC00693, Long Intergenic Non Protein Coding RNA 693; SPTSSB, Serine Palmitoyltransferase Small Subunit B; KCNIP3, Kv Channel Interacting Protein 3; MED12L, Mediator Complex Subunit 12 Like; KPNA1, Karyopherin Subunit Alpha 1; LCORL, Ligand Dependent Nuclear Receptor Corepressor Like; CLCN3, Chloride Voltage Gated Channel 3; RIMS1, Regulating Synaptic Membrane Exocytosis 1; PAM, Peptidylglycine Alpha Amidating Monooxygenase; C5orf24, Chromosome 5 Open Reading Frame 24; RPS12, Ribosomal Protein S12; FYN, FYN Proto Oncogene Src Family Tyrosine Kinase; TRIM40, Tripartite Motif Containing 40; GS1124K5.11, Uncharacterized GS1124K5.11; UBAP2, Ubiquitin Associated Protein 2; GBF1, Golgi Brefeldin A Resistant Guanine Nucleotide Exchange Factor 1; RNF141, Ring Finger Protein 141; FAM49B, Family With Sequence Similarity 49 Member B; SCAF11, SR Related CTD Associated Factor 11; FBRSL1, Fibrosin Like 1; MBNL2, Muscleblind Like Splicing Regulator 2; RPS6KL1, Ribosomal Protein S6 Kinase Like 1; CAB39L, Calcium Binding Protein 39 Like; CD19, CD19 Molecule; MIPOL1, Mirror Image Polydactyly 1; CHRNB1, Cholinergic Receptor Nicotinic Beta 1 Subunit; UBTF, Upstream Binding Transcription Factor; NOD2, Nucleotide Binding Oligomerization Domain Containing 2; GRN, Granulin Precursor; MED13, Mediator Complex Subunit 13; MEX3C, Mex 3 RNA Binding Family Member C; ASXL3, Additional Sex Combs Like 3 Transcriptional Regulator; DYRK1A, Dual Specificity Tyrosine Phosphorylation Regulated Kinase 1A; DNAH17, Dynein Axonemal Heavy Chain 17; CRLS1, Cardiolipin Synthase 1.

**Figure 1:**
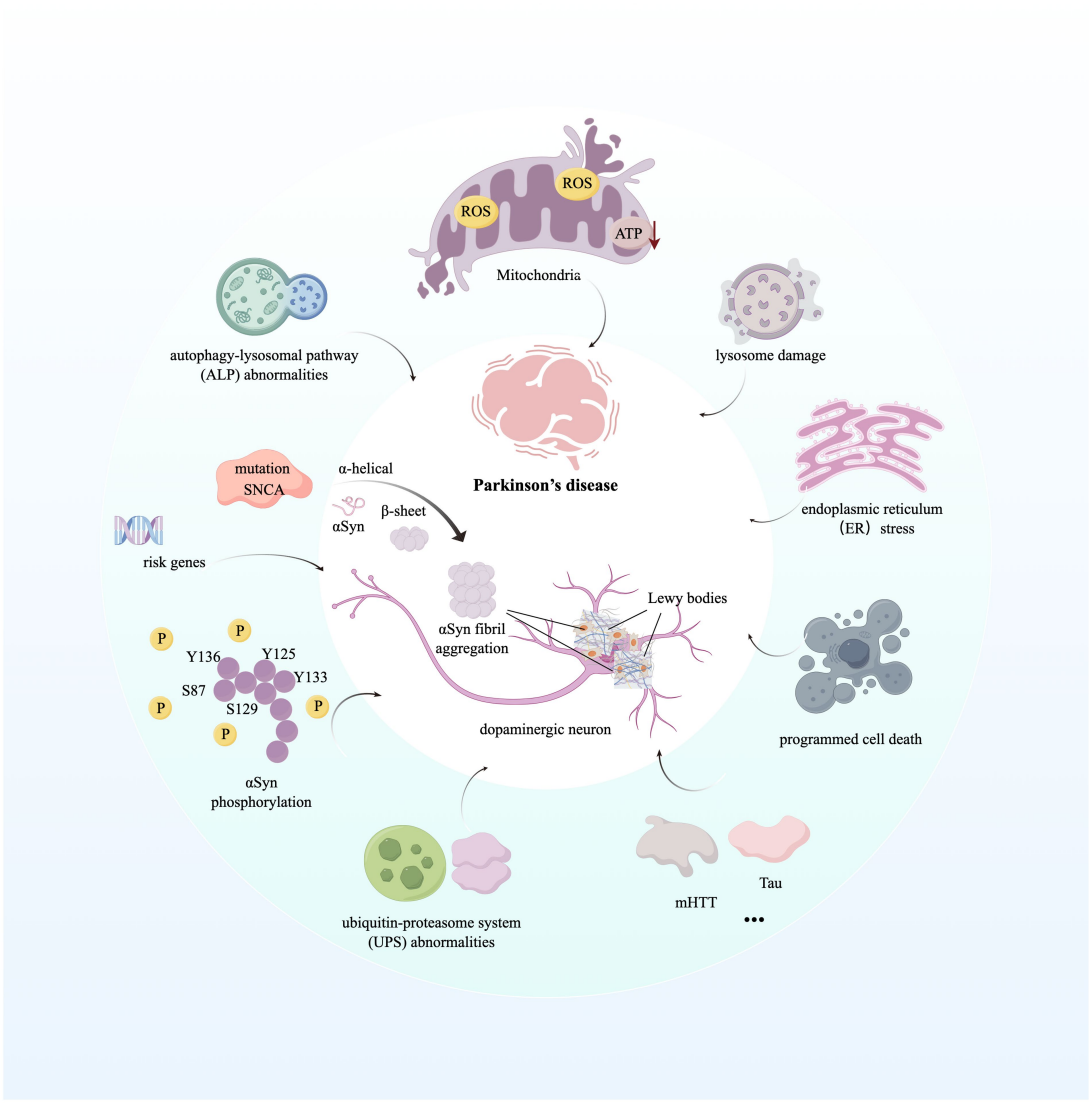
Molecular mechanisms contributing to Parkinson’s disease. The image illustrates the aggregation of the hallmark protein α-synuclein in Parkinson’s disease (PD) and the subsequent formation of Lewy bodies. Furthermore, it highlights the mechanisms of disease progression initiated by dysfunctions in various biological processes and organelles within the intracellular environment. For detailed descriptions, refer to the corresponding sections in the text. This figure was created using Figdraw. ROS, Reactive Oxygen Species; ATP, Adenosine Triphosphate; SNCA, Synuclein Alpha; mHTT, Mutant Huntingtin; Tau, Microtubule-Associated Protein Tau.

Understanding of altered genes related to PD onset and progression has provided greater insight into the molecular mechanisms that contribute to disease pathogenesis. Disruption of intracellular homeostasis, such as endoplasmic reticulum (ER) stress, impairment of mitochondrial and lysosomal function, and dysregulation of lipid metabolism and signaling between the ER and mitochondria, leads to the acceleration of *α*-Syn accumulation, Lewy body formation, and neuronal cell death (Bloem et al., 2021). Briefly, mitochondria form a dynamic, interconnected network with other cellular compartments, generating ATP and many biosynthetic intermediates (Nunnari and Suomalainen, 2012). Meanwhile, the dysfunction of mitochondria and altered mitochondrial structure are hallmarks of PD, leading to the production of reactive oxygen species (ROS), abnormal intracellular calcium levels, and reduced mitochondrial ATP generation, resulting in neural programmed cell death (PCD) (Poewe et al., 2017). Significantly, mutations in 11 genes reported in PD patients are known to impair mitochondrial quality and functions, such as cellular energy production through mitochondrial complex I activity inhibition (Morris et al., 2024; Schapira, 2012). In addition, the excessive production of ROS and dysfunction of mitochondria contribute to the formation of *α*-Syn aggregations (Picca et al., 2021). Besides, abnormalities in the autophagy-lysosomal pathway (ALP) and the ubiquitin-proteasome system (UPS) of PD patients lead to the dysregulation of selective and targeted degradation of abnormal or misfolded protein species, such as *α*-Syn, and cause an increased level of α-Syn aggregations (Ebrahimi-Fakhari et al., 2012). Similarly, overactivation of LRRK2 and VSP35 due to pathogenic mutation can excessively phosphorylate a series of Rab proteins, leading to the dysregulation of endocytosis and intracellular trafficking, finally causing deficiency in lysosomal function and aberrant cellular response to membrane damage under PD conditions (Nguyen et al., 2019). Furthermore, α-Syn aggregations can trigger innate immune response via binding to Toll-like receptors on microglial cells and peripheral monocytes, and then induce a specific adaptive T-cell response (Kouli et al., 2019; Sulzer et al., 2017). It is broadly hypothesized that α-synuclein aggregates, mitochondrial antigens, and even gut bacterial endotoxins facilitate innate and adaptive immune responses, hyper-activating neuroinflammatory pathways and neuronal toxicity among monocytes, neutrophils, microglia, pro-inflammatory Th1 and Th17 cells, and other immune cells, which enhance PD progression (Morris et al., 2024; Tansey et al., 2022). We have described molecular mechanisms contributing to PD in Figure 1.

For nearly as long as we have recognized PD, epidemiologic studies have suggested that extrinsic factors, including increasing age, male sex, environmental agent exposure, such as pesticide and chlorinated solvents exposure, unhealthy lifestyle, such as high dietary intake of dairy products, lower socioeconomic status, rural living, and traumatic brain injury (TBI), as well as intrinsic factors, including genetic predisposition, unusual metabolic states, and comorbidities such as diabetes, can be recognized as risk factors (Ben-Shlomo et al., 2024; Ascherio and Schwarzschild, 2016). While physical activity, smoking, higher serum urate, and caffeine exposure can be recognized as protective factors (Quik, 2004). Significantly, prevention in those at risk for PD because of genetic or environmental causes might be more effective than trials in people with established PD (Ben-Shlomo et al., 2024).

PD is strikingly heterogeneous according to the age of onset, clinical presentation, rate of progression, and treatment response (Tolosa et al., 2021). A recent study empirically identified the following common PD subtypes, including old age at onset and rapid disease progression subtype, young-onset PD (YOPD) and slow progression subtype where patients have a good response to dopaminergic medications, tremor-dominant phenotype subtype, and postural instability and gait difficulty (PIGD) subtype (Thenganatt and Jankovic, 2014). Meanwhile, the diagnosis of PD is based on history, typical asymmetric motor signs, no atypical features, and exclusion of alternative etiologies (Armstrong and Okun, 2020). However, the rate of misclassification and misdiagnosis of PD in routine clinical practice ranges from 15 to 24%, making it essential to gain more insights into the neuronal and molecular mechanisms underlying PD pathogenesis that can better identify specific biomarkers to categorize various PD phenotypes into subtypes and enhance diagnostic accuracy (Tolosa et al., 2021). A recent study put forward a biological classification of PD, which enables advances in developing new diagnostic criteria and precision medicine targeting disease-modifying therapies (Höglinger et al., 2024). At the clinicopathological level, the progression of PD is related to the proportion of SN neuron losses and the motor effects of loss of dopaminergic innervation of the basal ganglia (Kalia and Lang, 2015). Meanwhile, other factors, including mutations in specific genes, state of inflammation, and impairment of lysosomes also determine disease progression (Liu et al., 2016; Stoker et al., 2020; Tan et al., 2021; Liu et al., 2021).

## 3 Smoking is associated with a lower incidence of Parkinson’s disease

Although a series of advanced and symptomatic therapies for PD have emerged, no effective treatments are known to delay and halt its onset or slow its progression. For this reason, exploiting neuroprotective factors for PD can significantly reduce its risk. Although tobacco has a significant negative impact on the development of nervous structures, neurotransmission, and cognitive functions, and promotes the development of neurodegenerative diseases, insomnia, and cerebrovascular diseases, smoking is the most reproducible environmental association that has a neuroprotective effect on PD (Ascherio and Schwarzschild, 2016; Fratiglioni and Wang, 2000; Hajdusianek et al., 2021). Despite the fact that cigarettes contain over 60 well-established carcinogens, growing evidence suggests that smoking can be recognized as a protective environmental or lifestyle factor associated with lower risk of PD (Rose et al., 2024). However, it remains unclear how smoking could protect against PD. Multiple independent studies have proved that the relative risk of PD in smokers is significantly lower than in nonsmokers, also extending to a reduction in Lewy body formations and lower incidence of Lewy-related dementia (Hernan et al., 2002; Ritz et al., 2007; Scott et al., 2005; Tsuang et al., 2010; Morozova et al., 2008). Significantly, it has also been reported that there is an inverse association between parental smoking and risk of PD (O'Reilly et al., 2009). The relationship between smoking and PD risk is correlated with gender and illustrates the opposite trend observed for lung cancer (Morozova et al., 2008). In particular, individuals with moist smokeless tobacco (Snus) use have a 60% lower Parkinson’s disease risk compared with never-snus users (Yang et al., 2017). In addition, PD smokers are less likely to die from neurologic causes compared to PD nonsmokers, and smokers were observed to have a lower levodopa (L-dopa) equivalent dose, a proxy of disease severity (Yoon et al., 2023). Meanwhile, monoamine oxidase B (MAO B; EC 1.4.3.4) engages in the breakdown of dopamine, while the inhibition of MAO B is associated with enhanced activity of dopamine, as well as with decreased production of ROS. Research has proposed that the brains of living smokers show a 40% decrease in the level of MAO B relative to nonsmokers or former smokers (Fowler et al., 1996). In addition, PD patients without a regular smoking history have lower levels of striatal dopamine transporter (DAT) and dopamine levels in the synaptic gap compared to PD patients with a regular smoking history (Wang et al., 2022). Particularly, the protective effect of smoking on PD is dose-dependent (Tan et al., 2003). Mendelian randomization (MR) investigations and associated endeavors to eliminate reverse causality bias and tackle these concerns have revealed a consistent protective influence of smoking, and the continuation of smoking, on the risk of PD (Heilbron et al., 2021; Mappin-Kasirer et al., 2020; Dominguez-Baleon et al., 2021). In addition, investigations have suggested that individuals passively smoking also have protective effects on PD (Searles Nielsen et al., 2012). Interestingly, it has been reported that PD patients illustrated an increased ease of quitting smoking, which suggests a pathological potential correlation between addiction and PD (Ritz et al., 2014). Additionally, tobacco consumption changes the composition of the microbiota in the gut in a way that mitigates intestinal inflammation, and then leads to less misfolding of the protein *α*-Syn in enteric nerves, reducing the risk of PD by minimizing propagation of the protein aggregates to the central nervous system (CNS) (Derkinderen et al., 2014). However, the protective association between smoking and PD risk observed in epidemiological studies has not been replicated in clinical trials of nicotine monotherapy. Specifically, patients with PD who received transdermal nicotine therapy did not significantly improve motor manifestations, and nicotine treatment alone failed to slow disease progression in early PD (Wood, 2017; Oertel et al., 2023). Thus, while smoking may protect against PD through multi-component interactions, isolated nicotine administration lacks efficacy, highlighting the need to identify (1) optimal nicotine dosing regimens and (2) co-therapies mimicking smoke-derived neuroprotective synergism.

Tobacco or the products of its combustion in smoke are composed of numerous chemical compounds, making it crucial to uncover which molecular exposures among the myriad constituents are responsible for the protection of PD. Growing evidence indicates that carbon monoxide, MAO-B inhibitors, and especially nicotine play a major role in such protection. Carbon monoxide (CO) is an inorganic chemical compound that can bind with a multitude of hemoproteins, boasting at least 25 identified targets, including hemoglobin, myoglobin, neuroglobin, cytochrome c oxidase, cytochrome P450, soluble guanylyl cyclase, myeloperoxidase, and several ion channels, with dissociation constant values ranging from sub-nM to high μM (Derkinderen et al., 2014). At low concentrations, CO can be recognized as an endogenous signaling molecule with a diverse array of pathophysiological and pharmacological roles, involving immunomodulation, organ protection, and circadian rhythm regulation through activating cytoprotective molecular pathways (Chu et al., 2021). For example, CO is able to activate protective factors of PD, such as hypoxia-inducible factor 1*α* (HIF 1α) and nuclear factor erythroid 2-related factor 2 (Nrf2), inhibit poly (ADP-ribose) polymerase (PARP) cleavage, reduce caspase-3 activation, inhibit cell death, and activate the release of adenosine by astrocytes (Zhang et al., 2011; Stein et al., 2012; Choi et al., 2016; Queiroga et al., 2016; Wang et al., 2011). However, once the concentration is more than 50%, CO is subjected to cause irreversible damage to multiple brain regions (Choi et al., 2016). Of note, the concentration of hemoglobin-bound CO levels in smokers is threefold or fourfold higher than in nonsmokers (5–9% vs. 0–2%) (Stewart, 1975). Accordingly, investigations have suggested that CO possesses a neuroprotective role in PD models. For instance, a low CO dose treatment can reduce α-Syn aggregations and protect neurons in the SN from cell death in both a 1-methyl-4-phenyl-1,2,3,6-tetrahydropyridine (MPTP) mouse model and an adeno-associated virus (AAV) α-synuclein rat model (Rose et al., 2024). In addition, CO can prevent apoptosis of hydroxydopamine (6-OHDA)-treated C6 glioma cells by decreasing the Bax/Bcl2 ratio and caspase 3 activity, and increasing phosphorylation of Nrf2 (Moon et al., 2018). Besides, heme oxygenase (HO-1) is the primary inducible enzyme that mediates the cleavage of heme, a molecule with proinflammatory and prooxidant properties, to generate endogenous CO, which is ubiquitously expressed and upregulated with Nrf2 in response to stress (Silva et al., 2020; Dwyer et al., 1995). In the SN of idiopathic PD patients, the expression of neuronal HO-1 is upregulated, which is localized in the peripheries of Lewy bodies (Schipper et al., 1998). Upregulation of HO-1 expression has a neuroprotective effect in 1-methyl-4-phenylpyridinium (MPP+) and MPTP PD toxin models, such as alleviating proinflammatory responses and the release of inflammatory cytokines, reducing dopamine cell loss, and increasing neurotrophic factors (Hung et al., 2008; Youn et al., 2014; Yamamoto et al., 2010). However, the association between CO in cigarette smoke and PD risk needs more epidemiological investigations. Monoamine oxidase (MAO) is a riboflavin protein distributed on the outer membrane of mitochondria, which catalyzes the oxidative deamination of neurotransmitters such as dopamine, phenethylamine, 5-hydroxytryptamine, and norepinephrine (Tripathi and Ayyannan, 2019). MAO has 2 isoenzymes: MAO-A and MAO-B, and MAO-B inhibitors inhibit MAO activity in the brain, block dopamine catabolism, enhance dopamine signaling, and selectively enhance dopamine levels at the synaptic cleft (Chen and Swope, 2005). MAO-B inhibitors have been identified in tobacco smoke, such as naphthoquinones, 2-napthylamine, and norharman, and have been recognized as a protective factor of PD (Reniers et al., 2011; Sari and Khalil, 2015). The clinical treatment of PD using MAO-B inhibitors can improve motor functions and non-motor functions and delay the use of L-dopa. Besides, smokers have been shown to have significantly reduced brain MAO-B binding sites compared to nonsmokers (Fowler et al., 1996). Besides, investigations have proved that MAO-B inhibitors can reduce dopaminergic cell death by inhibiting the oxidation of MPTP and formation of MPP+ by MAO-B (Chan et al., 2018). MAO-B inhibitors can also promote *α*-Syn to form cyclic or dimeric structures and prevent it from forming sheet-like structures and linear aggregates, thereby potentially reducing the toxic effects associated with α-Syn aggregation (Kakish et al., 2015). Particularly, nicotine has been the dominant candidate proposed to underlie the reduced risk of PD among smokers for nearly 50 years.

## 4 Neuroprotection of nicotine in Parkinson’s disease

Nicotine is a natural alkaloid mainly found in the tobacco plant (*Nicotiana tabacum*). It is recognized for its psychoactive effects, particularly as a stimulant of the central nervous system, with a chemical structure that includes a pyridine ring and a pyrrolidine ring, resulting in the molecular formula C₁₀H₁₄N₂ and a molecular weight of 162.23 g/mol (Le Foll et al., 2022). Nicotine is linked to the harmful effects of tobacco smoking, such as cardiovascular disease and various cancers. Long-term nicotine use can lead to dependence and withdrawal symptoms, highlighting the contrast between its therapeutic benefits and associated risks. However, nicotine also acts as an agonist on nicotinic acetylcholine receptors (nAChRs), which play a key role in regulating neurotransmitter release, neuronal excitability, and cognition (Dineley et al., 2015). Hence, it is also being researched for its potential neuroprotective properties, especially in neurodegenerative conditions like PD (Ascherio and Schwarzschild, 2016).

Nicotine has neuroprotective capabilities largely attributed to its ability to initiate protective signaling transduction that enhances dopamine release and reduces neurotoxicity. Research shows that nicotine activates nAChRs on dopaminergic neurons, which are crucial for regulating neurotransmitter release and neuronal excitability (Quik et al., 2008). Studies have demonstrated that nicotine administration can mitigate the degeneration of dopaminergic neurons triggered by 6-OHDA, rotenone, or MPTP by decreasing the level of α-Syn (Ullah et al., 2024; Ullah et al., 2024; Fares et al., 2023; Elgayar et al., 2022; Mouhape et al., 2019). In PD mouse models, nicotine can improve hyposmia via the prok2R/Akt/FoxO3a signaling pathway (Guo et al., 2024). and significantly improve motor impairment through activating JNK and ERK signaling pathways in the nigra-striatum related brain regions (Ruan et al., 2023). Nicotine also enhances dopaminergic projections and L-dopa sensitivity (Oldehinkel et al., 2022; Ma et al., 2017). Research indicates that nicotine and its analogs activate the PI3K/Akt pathway, promoting neuron survival, cell growth, resistance to apoptosis, and reducing oxidative stress (Rojas-Rodriguez et al., 2020; Kume and Takada-Takatori, 2018). Nicotine exerts a neuroprotective effect by degrading Sirtuin 6 (SIRT6) (Nicholatos et al., 2018). and improving mitochondrial quality and function, which plays a fundamental role in the onset and progression of PD. Specifically, nicotine can exert antioxidant effects by enhancing mitochondrial function and reducing lipid peroxidation (Godoy et al., 2018; Bridge et al., 2004). Additionally, research demonstrates that nicotine protects neurons from oxidative stress by activating nAChRs and reducing the production of reactive oxygen species (ROS) (Ciobica et al., 2012; Cormier et al., 2003). Nicotine also enhances mitochondrial function by activating nAChRs, increasing neuron survival rates (Cormier et al., 2003). and protects astrocytes against apoptosis by preserving mitochondrial membrane potential and inhibiting the mitochondrial apoptotic pathway (Liu et al., 2015). By activating the PINK1 (PTEN-induced kinase 1)/Parkin (PDR-1) pathway, nicotine enhances mitochondrial quality and reduces oxidative stress in dopaminergic neurons (Nourse et al., 2021). The inflammatory response significantly contributes to the progression of PD, and recent research suggests that nicotine may exert anti-inflammatory effects by inhibiting microglia and astrocyte activation and reducing the production of pro-inflammatory cytokines (Cafe-Mendes et al., 2017; Cui and Li, 2010). Nicotine inhibits the TLR4/NF-κB signaling pathway through the activation of nAChR, reducing the production of pro-inflammatory cytokines (Cafe-Mendes et al., 2017). And protects neurons against ER stress (Srinivasan et al., 2016). Research has also shown that nicotine influences the gut microbiota, which is associated with the risk of PD (Scheperjans et al., 2015). Furthermore, nicotine’s neuroprotective mechanisms extend to influencing neurotrophic factors, particularly brain-derived neurotrophic factor (BDNF), which supports neuronal health and resilience in adverse conditions (Huang et al., 2020). Nicotine can protect neurons in Parkinson’s disease by managing neurotransmitter release and enhancing neural plasticity. It activates nAChRs, increasing dopamine release and improving motor function in PD patients (Kirch et al., 1988). In addition to its effects on dopamine, nicotine enhances neuronal function by regulating the release of other neurotransmitters, such as glutamate and gamma-aminobutyric acid (GABA) (Lai et al., 2023; Kunisawa et al., 2018). A comprehensive analysis of neurotransmitter alterations within the hippocampal region following nicotine administration provides novel insights into the management of PD (Zhu et al., 2024). Nicotine’s multifaceted approach includes balancing excitatory and inhibitory signals, reducing oxidative stress and inflammation, and enhancing neuronal growth factors, suggesting its potential role in addressing the complex neurological challenges of PD.

The link between nicotine and Parkinson’s disease (PD) has been extensively investigated through epidemiological studies and clinical trials. These trials primarily focus on nicotine replacement therapies (NRT), which aim to reduce symptoms and improve the quality of life for individuals with PD. Clinical studies indicate that nicotine NRT may reduce motor symptoms such as tremors, rigidity, and bradykinesia (Quik et al., 2015). Notably, a randomized controlled trial (RCT) demonstrated that patients using a nicotine patch showed significant improvement in motor function and overall symptom management compared to the placebo group. A review highlighted the safety and efficacy of low-dose nicotine treatments, suggesting a promising therapeutic avenue for future studies (Oertel et al., 2023).

While the aforementioned data is promising, using nicotine clinically for PD presents challenges, including toxicity, craving behaviors, and variations in individual responses. More carefully designed studies are needed to thoroughly evaluate various dosages, treatment methods, and modes of administration. As research progresses, it becomes clear that a tailored approach to nicotine use is essential for effectively managing PD.

## 5 Molecular function and structure of nicotinic acetylcholine receptor subtypes and its role in Parkinson’s disease

nAChRs are a family of ion channels that are extensively distributed throughout the central nervous system (CNS). Their primary function is to mediate responses to the endogenous neurotransmitter acetylcholine (ACh) and exogenous nicotine, thereby influencing downstream biological processes through neural signal conduction.

nAChRs are macromolecular complexes of receptors and channels with a pentameric structure. Their main structural components include subunits with ACh binding sites, oriented with extracellular receptor domains that create a central pore channel, along with several hydrophobic transmembrane domains (Albuquerque et al., 2009). The diversity in structure due to various subunit combinations underlies the functional diversity of nAChRs. This functional diversity is reflected in physiological and pharmacological characteristics, such as the strength of membrane depolarization, the kinetics of gated activation, the rate of desensitization and resensitization, and the magnitude of ionic signaling. Generally, nAChR subtypes can be differentiated based on their subunit assemblies. Naturally expressed nAChRs are assembled as either homomers or heteromers from a repertoire of at least 17 subunits, including *α*1–α10, *β*1–β4, *γ*, *δ*, and *ε* (Lukas et al., 1999). Mammalian neuronal nAChRs also maintain a pentameric structure but have relatively fewer subunits available for assembly, primarily including the α (α2-α7, α9, and α10) and β (β2-β4) classes (Albuquerque et al., 2009). Different nAChR subtypes exhibit cell-specific expression due to differences in subunit gene promoters, subunit asymmetry and lateralization, varied synaptic structures, and differing biological contexts. This theoretically results in a wide range of biological functions and applications linked to the diverse array of subtype varieties. In practice, the assembly and stoichiometry of subunits are stringently regulated. For instance, β3 and α5 subunits cannot form nAChRs independently or with any other single type of subunit; they can, however, integrate into binary complexes to form ternary assemblies, and even contribute to the formation of quaternary structures such as α4α6β2β3 (Wu and Lukas, 2011).

In the mammalian brain, the predominant high-affinity nicotine nAChR subtypes consist of α4 and β2 subunits, as well as α7 homomeric subunits (Albuquerque et al., 2009; Dani et al., 2000; Mansvelder and McGehee, 2000). These subtypes are implicated in nicotine dependence and various neurodegenerative disorders. When nicotine first reaches the midbrain dopamine (DA) regions, it engages high-affinity α4β2* nAChRs and similar subtypes, thereby enhancing neurotransmitter release and promoting neuronal activity through presynaptic nAChRs (Albuquerque et al., 1997; Alkondon et al., 2007). Additionally, the binding of nicotine induces conformational alterations in nAChRs and/or associated proteins, which activate various intracellular signaling pathways and regulate gene expression (Schaal and Chellappan, 2014). nAChRs in the brain not only mediate neurotransmission but also modulate neuronal function and status via chemical messengers, thereby influencing factors such as peptide neurotransmission, the cytoskeleton, protein kinases, and calcium ions (Mulle et al., 1992; Vernino et al., 1992; Amador and Dani, 1995) For example, calcium regulation mediated by nAChRs initiates the release of mitogenic factors through elevated cytosolic calcium levels, which in turn activates signaling cascades involved in cell proliferation, migration, angiogenesis, and the inhibition of apoptosis (Ambrosi and Becchetti, 2013). Additionally, certain nAChRs are associated with the structure and maintenance of neurites and synapses, thereby participating in the regulation of neuronal viability and death. Their functions are broadly involved in the modulation of cognitive function, anxiety, depression, Alzheimer’s disease, Parkinson’s disease, pain, and epilepsy, among other pathological conditions (Wu and Lukas, 2011; Kutlu and Gould, 2015; Higley and Picciotto, 2014).

The function and alteration of nAChRs are involved throughout the pathological cycle of Parkinson’s disease (PD), and their distribution in the human brain is closely associated with the progression of PD. The assembly capability of nAChR homomers and heteromers declines with human aging, and this decline is further exacerbated during the progression of PD (Gotti et al., 2006). In normal brain tissue, the distribution of nAChRs aligns with that of high-affinity nicotinic ligands, with nAChRs typically distributed along nerve fiber tracts. The loss of nigrostriatal dopaminergic markers is closely linked to nAChR alterations and may serve as an early indicator of nigrostriatal degeneration (Pimlott et al., 2004). According to the dopamine-acetylcholine balance hypothesis, dopamine depletion leads to severe deterioration of basal ganglia circuit dynamics, resulting in overactivation of the cholinergic system and subsequent motor and cognitive impairments. In the early stages of PD, there is upregulation of cholinergic activity at the striatal and cortical levels, with an increased density of nAChRs potentially serving as a compensatory mechanism to sustain dopaminergic tone. In PD patients, nAChR density is significantly higher in the putamen, insular cortex, and supplementary motor area compared to the caudate nucleus, orbitofrontal cortex, and middle temporal gyrus. There is an association between the use of anticholinergic drugs and cognitive decline in Parkinson’s disease (Isaias et al., 2014).

As regulators of neural and metabolic changes, nAChRs play a critical role in brain function and metabolism (Guan, 2024). In patients with Parkinson’s disease (PD), characterized by the selective degeneration of dopaminergic neurons, nAChRs can inhibit the development of neurotoxicity induced by rotenone and 6-hydroxydopamine, and 1-methyl-4-phenyl-1,2,3,6-tetrahydropyridine (MPTP) via inhibiting apoptosis of neurons, mitigation of oxidative stress and reduction of neuroinflammation (Kawamata et al., 2012). There are significant reductions in the expression of *α*4 and α7 nAChR subunits in the cerebral cortex of PD patients, along with the selective loss of nAChRs containing α3 and β2 subunits. Additionally, in PD patients, striatal deficits in α6 and β3 subunits are often more pronounced than those in α4 and β2 (Burghaus et al., 2003; Martin-Ruiz et al., 2000).

**Figure 2:**
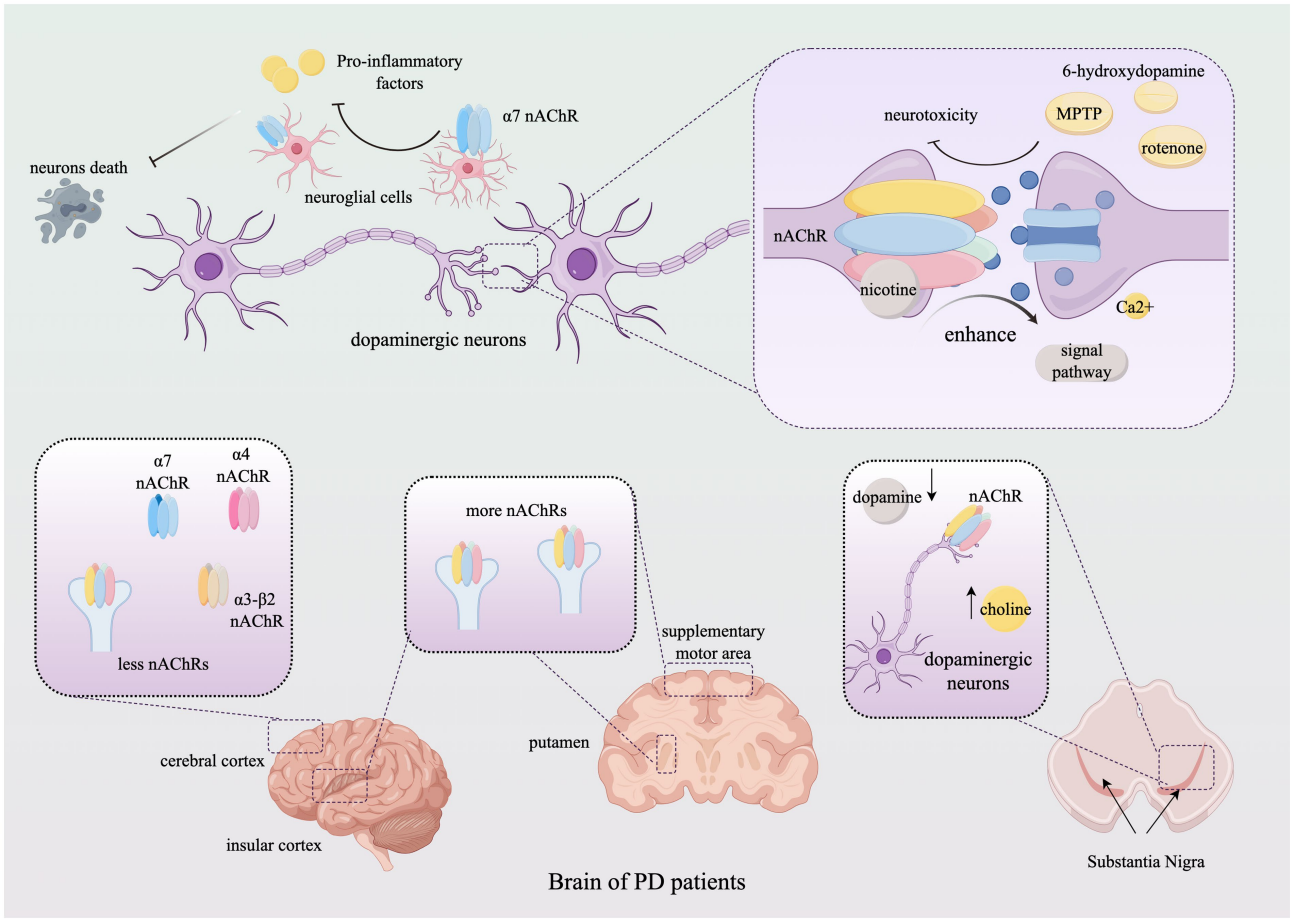
The role of nicotinic acetylcholine receptors in Parkinson’s Disease. The figure illustrates the alterations in the subtype distribution of nAChRs within the brains of PD patients throughout the progression of the disease and elucidates how nAChRs mediate neuroprotective effects by modulating neuronal behavior through neurotransmitter signaling. For detailed descriptions, refer to the corresponding sections in the text. This figure was created using Figdraw. nAChR, Nicotinic acetylcholine receptor; MPTP, 1-methyl-4-phenyl-1,2,3,6-tetrahydropyridine; Ca, calcium.

Neurological lesions are often accompanied by neuroinflammation, with excessive inflammation potentially causing damage to neurons and myelinated glial cells. Studies have shown that acetylcholine and its receptors play a role in regulating central and peripheral inflammation, suggesting that targeting neuroinflammation could reduce brain injury and promote neuronal survival. In particular, the α7 nicotinic receptor plays a significant role in regulating neuroinflammation in astrocytes and microglia. For example, the anti-inflammatory and neuroprotective properties of GTS-21, a selective agonist of α7 nAChR, have been demonstrated in mouse models of Parkinson’s disease (PD). In these models, GTS-21 mitigates dopaminergic neuronal death and inhibits microglial activation and the expression of pro-inflammatory factors. Moreover, the α4β2 and α7 nAChR subtypes and/or receptor subunits have demonstrated potential in improving cognitive impairment and providing neuroprotection in the brain (Piovesana et al., 2021; Park et al., 2022). Additionally, neuronal nicotinic acetylcholine receptors are located in the outer mitochondrial membrane, where they modulate mitochondrial dysfunction and thus participate in the regulation of neuroinflammation and disease progression (Skok, 2022). We describe the role of nAChRs in the development of Parkinson’s disease (PD) in Figure 2 (Table 2).

**Table 2:** Mechanisms of nicotine and nAChRs in Parkinsonian animal models.

PD Model	Mechanisms of nicotine	nAChRs subunits	Mechanisms	References
MPTP-induced mice	Neuroprotection via DA neuron survival	α4β2, α7	PI3K/Akt, Bcl-2/Bax facilitationf	Wiest et al. (2020)
6-OHDA-lesioned rats	Anti-inflammatory	α7	NF-κB/STAT3 inhibition	Yang et al. (2021) and Chen et al. (2023)
α-syn transgenic mice	Reduced α-syn aggregation	α7	mTOR/ULK1 pathway facilitation	Shen et al. (2024)
AAV-mediated SNCA mice	Enhanced synaptic plasticity	α6β2	ERK/CREB phosphorylation	Dickinson et al. (2008)

MPTP, 1-Methyl-4-phenyl-1,2,3,6-tetrahydropyridine; 6-OHDA, 6-Hydroxydopamine; nAChR, Nicotinic acetylcholine receptor; α-syn, α-synuclein; DA, Dopaminergic; PI3K/Akt, Phosphatidylinositol 3-kinase/Protein kinase B; NF-κB, Nuclear factor kappa B; mTOR, Mechanistic target of rapamycin; ULK1, Unc-51-like kinase 1; ROS, Reactive oxygen species; SOD, Superoxide dismutase; GSH, Glutathione; Nrf2/ARE, Nuclear factor erythroid 2-related factor 2/Antioxidant response element; AAV, Adeno-associated virus; SNCA, α-synuclein gene; ERK, Extracellular signal-regulated kinase; CREB, cAMP response element-binding protein.

## 6 Conclusion

Parkinson’s disease (PD) has a profound impact on modern society. Despite considerable progress in understanding its pathobiology, the complex etiology of the disease and challenges in clinical diagnosis and treatment accuracy continue to pose significant challenges. To enhance our understanding of PD’s pathological mechanisms and its challenging clinical landscape, we systematically review the comprehensive characteristics of PD and establish a link between smoking behavior and PD incidence through epidemiological analysis. The potential mechanisms by which smoking influences PD primarily involve the neuroprotective chemical constituents of cigarettes, their dosages, and their roles in neurobiological processes. Nicotine and carbon monoxide (CO) in cigarettes exhibit neuroprotective capabilities, supporting the rationale behind this associative model. Specifically, nicotine confers neuroprotection by enhancing dopamine activity and modulating reactive oxygen species (ROS), while CO exerts neuroprotective effects by regulating antioxidant responses and inhibiting apoptosis. In addition, *In vitro* studies using human dopaminergic neurons derived from induced pluripotent stem cells (iPSCs) have provided critical mechanistic insights:(1) nicotine (10 μM) activates α7 nAChRs to reduce α-synuclein oligomerization and mitochondrial fission in PD patient-derived neurons, effects blocked by the selective antagonist α-bungarotoxin; (2) α6β2 nAChR activation in SH-SY5Y cells promotes neurite outgrowth via ERK/CREB phosphorylation, which increases evidence of the neuroprotection of nicotine (Bono et al., 2019; Collo et al., 2013).

However, it is critical to acknowledge that most preclinical evidence supporting nicotine’s therapeutic potential originates from acute toxin-based models (e.g., MPTP or 6-OHDA-induced neurodegeneration in rodents/non-human primates). While these models provide mechanistic insights, they oversimplify human PD pathology by (1) lacking progressive α-synuclein aggregation, (2) ignoring aging-related metabolic and epigenetic changes, and (3) failing to mimic the decades-long prodromal phase of human PD. Despite robust evidence from preclinical studies demonstrating nicotine’s neuroprotective effects in animal PD models, recent transdermal nicotine trials in early-stage PD patients have failed to replicate these benefits. For instance, a phase II clinical trial revealed no significant slowing of disease progression with nicotine treatment, highlighting a critical translational gap (Oertel et al., 2023). This discrepancy may arise from (1) interspecies differences in nAChR subtype expression and metabolic pathways; (2) failure to mimic chronic, low-dose nicotine exposure patterns observed in smokers; (3) the inability of animal models to fully recapitulate the multifactorial pathology of human PD. Addressing these limitations, future preclinical studies should prioritize longitudinal designs with nAChR subtype-specific agonists/antagonists and genetically engineered models incorporating humanized nAChR subunits.

Notably, other studies points out that a gender differences in the correlation between smoking and the risk of Parkinson’s disease (Allam et al., 2007). Large-scale cohort studies reveal that male smokers have a 30–40% reduced risk of PD compared to non-smokers, whereas the protective effect in female smokers is attenuated or absent. Potential explanations include (1) Estrogen-mediated modulation of nAChR expression in females (2) Sex-specific differences in nicotine metabolism (3) Interactions between smoking and hormonal replacement therapy in postmenopausal women. These findings underscore the need for gender-stratified analyses in future epidemiological and therapeutic studies.

Nicotinic acetylcholine receptors (nAChRs), as nicotine targets, play a crucial role in neuronal function and PD progression through their multifunctional subtypes composed of various subunits. Notably, while *α*4β2 and α7 nAChRs are implicated in dopamine neuron survival via PI3K/Akt and NF-κB pathways, their exact molecular interplay in PD remains speculative due to a paucity of clinical biomarker studies. Importantly, the therapeutic optimism derived from toxin models may not translate to human PD if nAChR signaling is merely compensatory in acute injury rather than causative in chronic neurodegeneration. Correlating cerebrospinal fluid (CSF) nAChR-related proteins (e.g., lynx1 or SLURP-1) with PD progression in ongoing nicotine trials could bridge this knowledge gap. In addition, this review establishes a framework linking smoking behavior, nicotine, and nAChRs, evaluating their plausibility and research potential in PD treatment. Our goal is to provide new perspectives for PD therapeutic research.

Future research should concentrate on (1) elucidating nAChR roles in genetic PD subtypes (e.g., GBA or SNCA mutation carriers), (2) developing disease-modifying strategies targeting nAChR-ligand interactions beyond symptomatic relief, and (3) integrating multi-omics approaches to identify nAChR-associated biomarkers predictive of therapeutic response. Additionally, the relevance of this emerging direction within the traditional PD research framework should be articulated, with a comprehensive analysis of nAChRs’ roles considering genetic, environmental, and molecular factors. To avoid over interpretation of preclinical data, we emphasize the need for rigorous reverse translation: validating mechanistic hypotheses from animal models in human biospecimens. We believe that this review will contribute to a more thorough understanding of PD pathogenesis and promote the development of personalized medicine. By effectively integrating advances in fundamental research and clinical applications, we aspire to achieve more effective PD diagnostic and therapeutic strategies in the near future.
